# Gas Chromatography–Mass Spectrometry Analysis of *Artemisia judaica* Methanolic Extract: Chemical Composition, Radical Scavenging Potential, Bioherbicidal Activity, and Dengue Vector Control

**DOI:** 10.3390/ijms26157355

**Published:** 2025-07-30

**Authors:** Naimah Asid H. Alanazi, Amani Alhejely, Sultan Mohammed Areshi, Hanan K. Alghibiwi, Samiah A. Alhabardi, Mohammed A. Akeel, Amal Naif Alshammari, Sarah Mohammed Alrajeh, Gadah A. Al-Hamoud, Salama A. Salama

**Affiliations:** 1 Department of Biology, College of Sciences, University of Ha’il, Ha’il 53962, Saudi Arabia; n.alenezy@uoh.edu.sa; 2 Biology Department, Darb University College, Jazan University, Jazan 45142, Saudi Arabia; alhejely@jazanu.edu.sa (A.A.); ashammari@jazanu.edu.sa (A.N.A.); 3 Biology Department, College of Science, Jazan University, P.O. Box 114, Jazan 45142, Saudi Arabia; sareshi@jazanu.edu.sa; 4 Pharmacology and Toxicology Department, College of Pharmacy, King Saud University, Riyadh 11451, Saudi Arabia; halghibiwi@ksu.edu.sa; 5 Department of Pharmaceutics, College of Pharmacy, King Saud University, Riyadh 11451, Saudi Arabia; salhabardi@ksu.edu.sa (S.A.A.); salrajieh1@ksu.edu.sa (S.M.A.); 6 Human Anatomy Department, Faculty of Medicine, Jazan University, Jazan 45142, Saudi Arabia; m.akeel@jazanu.edu.sa; 7 Department of Pharmacognosy, College of Pharmacy, King Saud University, P.O. Box 2457, Riyadh 11451, Saudi Arabia; galhamoud@ksu.edu.sa; 8 Zoology Department, Faculty of Science, Damanhur University, Damanhur 22511, Egypt

**Keywords:** *Artemisia judaica*, Asteraceae, volatile constituents, phytotoxicity, free radical scavenging

## Abstract

Today’s primary challenges include identifying efficient, affordable, and environmentally sustainable substances to serve as raw materials in industrial, agricultural, and medicinal applications. This study aimed to evaluate the chemical composition and biological properties (namely antioxidant and allelopathic activities) of the methanolic extract derived from the above-ground portions of *Artemisia judaica* collected in Jazan, Saudi Arabia. GC-MS was used to evaluate the chemical composition of the methanolic extract derived from *Artemisia judaica*. GC-MS analysis revealed a total of 22 volatile compounds in the extract. The most prominent compounds identified were 2-ethylhexanoic acid, 5-hydroxy-6-(1-hydroxyethyl)-2,7-dimethoxynaphtho-quinone, and piperitone. The extract demonstrated strong antioxidant activity in both the DPPH and ABTS radical scavenging assays, comparable to the standard antioxidant ascorbic acid. The IC_50_ value for the extract was 31.82 mg/mL in the DPPH assay and 39.93 mg/mL in the ABTS testing. Additionally, the extract exhibited dose-dependent inhibition of seed germination, root growth, and shoot growth of the weed *Chenopodium murale* in allelopathic bioassays. The most significant suppression was observed in shoot growth with an IC_50_ value of 45.90 mg/mL, which was lower than the IC_50_ values for root development and seed germination of *C. murale*, recorded at 56.16 mg/mL and 88.80 mg/mL, respectively. Furthermore, the findings indicated that methanolic extracts had significant lethal toxic effects on the life cycle of *Aedes aegypti*. Future research will focus on extracting uncontaminated substances and evaluating the biological effects of each specific constituent.

## 1. Introduction

The tribe *Anthemideae*, part of the family Asteraceae (also known as *Compositae* Giseke), includes a diverse array of flowering plants with numerous ethnomedicinal uses [1,2]. A prime example is the genus *Artemisia* L., which comprises aromatic herbs and shrubs. This genus contains over 500 species and is widely distributed in the northern temperate regions of the world [3,4]. Among these is the desert chemotype *Artemisia judica* L. (*A. judaica*), which belongs to the *Anthemideae* tribe [5]. *A. judaica* is a perennial, bushy, fragrant plant characterized by woody bases and a coating of wooly hairs [6,7,8]. Its leaves are grayish and short and feature spherical, densely packed heads with tubular flowers. Traditional medicines containing *A. judaica* are known to possess anti-inflammatory, antibacterial, antiviral, wound-healing, anti-diabetic, and fever-reducing properties, akin to those found in other *Artemisia* species [5,6,7,8]. This plant has been widely utilized by traditional medicine practitioners in the Arabian Desert due to its anthelmintic, antibacterial, anti-inflammatory, and analgesic effects [6,7,8]. Additionally, it is used to alleviate external injuries, coughing, diarrhea, ear infections, scorpion bites, and snake bites.

Chemical analysis of *A. judaica* revealed the presence of flavonoids, sesquiterpene lactones, triterpenes, and essential oil [6,8,9]. Additionally, certain plant genotypes have been found to contain relatively high concentrations of various chemical constituents, such as camphor, ethyl cinnamate, and spathulenol [8]. The essential oil from *A. judaica* has been associated with a variety of biological activities, including anticancer activity [10,11], antimicrobial activity [7,9], antioxidant activity [7,12], anti-inflammatory activity [7,13], and wound healing [7]. The flavonoid from *A. judaica* displayed anticancer properties [14,15]. Artemisinin sesquiterpene lactone from *A. judaica* displayed antimicrobial activity [16].

Solvents significantly influence the extraction process, impacting both the quantity and characteristics of secondary metabolites found in medicinal plants [17,18,19]. From a biological perspective, water and alcoholic extracts of *A. judaica* considerably reduced blood sugar levels [20,21]. The hexane and chloroform extracts exhibited antimicrobial properties, whereas the chloroform extract also displayed anticancer properties [9]. The ethanol extract exhibited antioxidant properties [18]. The 70% aqueous ethanol extract of *A. judaica* demonstrated higher potential and specific antiangiogenic activities [6]. Ethyl acetate and methyl alcohol extracts exhibited antimicrobial properties [19].

Traditional medicine derived from medicinal plants in the Kingdom of Saudi Arabia highlights a significant connection to natural remedies, health, diet, and folk healing practices, all of which are essential to its unique culture. This study seeks to examine the antioxidant properties, bioherbicidal effects, and chemical composition of the above-ground parts of *A. judaica* obtained from Saudi Arabia, utilizing an 80% aqueous methanol extract.

## 2. Results and Discussion

### 2.1. GC-MS Analysis

**Figure 1:**
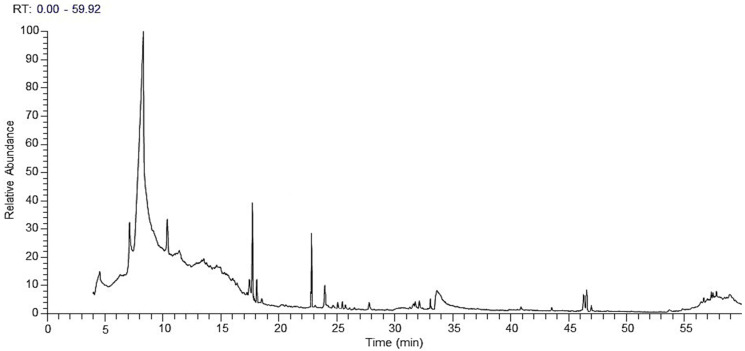
Chromatogram displaying key volatile components of *Artemisia judaica* extract obtained through GC-MS analysis.

The GC-MS analysis was conducted to identify the volatile components of the methanol extract of *A. judaica* [17,22,23,24]. The chromatogram presented in Figure 1 shows the relationship between the retention times of specific detected components and the relative quantities of different components identified from the extracted plant material. The findings in Table 1 indicated that 22 volatile components were successfully identified by the GC-MS method over a retention time of 59.92 min, accounting for 100% of the total MeOH extract of *A. Judaica*. The main component identified was 2-ethylhexanoic acid, which made up 28.41% of the extract and eluted at 8.34 min. Other substances were later categorized based on higher compositional percentages, including 5-hydroxy-6-(1-hydroxyethyl)-2,7-dimethoxynaphtho-quinone (17.54%) and piperitone (15.74%), which were eluted at 17.73 and 22.79 min, respectively. These compounds constituted 61.69% of the total identified compounds (Table 1). Additionally, other chemical constituents were identified with notable percentages, such as glycerol (7.09%), octanoic acid (6.30%), methyl 4,6-tetradecadiynoate (4.35%), and methyl 5,7-hexadecadiynoate (3.53%). The results also highlighted other components with relatively low composition percentages, as indicated in Table 1.

As verified from Table 1, the volatile components of *A. judaica* extracted with methanol were 22 components from which seven were identified as oxygenated hydrocarbons, nine were identified as fatty acids and their derivatives, and six were identified as terpenes. The primary class identified consists of fatty acids and their derivatives, accounting for 45.54% of the total area, with 2-ethylhexanoic acid being the most prominent compound. These compounds eluted within a range of 7.14 to 46.96 min. Hydrocarbons constitute 30.25% of the total, with 5-hydroxy-6-(1-hydroxyethyl)-2,7-dimethoxynaphthoquinone as the most notable compound, eluting between 10.39 and 57.3 min. Terpenes make up 24.21% of the total, with piperitone identified as the most prominent compound, eluting between 22.79 and 47.02 min.

The diversity and quantity of components extracted in this investigation closely resemble those reported in previous studies. El-Afify et al. identified 2-ethylhexanoic acid as the predominant phytochemical in the xero-halophyte Zygophyllum coccineum [25]. Hernández-Aparicio et al. observed that tomato plants emit varying quantities of 2-ethylhexanoic acid [26]. Xu documented the presence of 5-hydroxy-6-(1-hydroxyethyl)-2,7-dimethoxynaphtho-quinone in mangrove-associated microbes [27]. The findings for Achillea biebersteini [28] and Mentha pulegium [29] confirm that piperitone is a major component. Additionally, three phytoconstituents—octanoic acid, 9(Z)-octadecenoic acid-methyl ester, and methyl 4,6-tetradecadiynoate—have been identified in other plants, including Datura stramonium [30], Azadirachta indica, Mangifera indica [31], and Citrus reticulata [32].

### 2.2. Radical Scavenging Activity of A. judaica MeOH Extract

The antioxidant activity of *A. judaica* was evaluated using the DPPH and ABTS methods, comparing the results to those obtained with ascorbic acid, a commonly used antioxidant (Table 2). The findings revealed that DPPH radical scavenging activity increased with higher concentrations of MeOH extract. Especially within the MeOH extract concentration range of 5 to 50 mg/mL, the DPPH color intensity decreased by 9.97% and 75.41%, respectively. In contrast, at the same extract concentrations, the ABTS color intensity was reduced by 7.65% and 60.12%, respectively. Additionally, at ascorbic acid concentrations of 1 and 20 mg/mL, the DPPH and ABTS color intensities decreased by 1.74% and 68.66%, respectively (Table 2). Thus, this extract functions as a dose-dependent scavenger of free radicals.

The aggregate results indicate that *A. judaica* extract possesses a notable DPPH free radical scavenging capacity, with an IC_50_ value of 31.82 mg/mL. Additionally, this extract demonstrates a significant ability to scavenge ABTS radicals, shown by its IC_50_ value of 39.93 mg/mL. In comparison, ascorbic acid, a reference antioxidant, displays lower IC_50_ values of 11.74 mg/mL for DPPH and 13.05 mg/mL for ABTS.

Zheng et al. (2016) proposed the one-step hydrogen atom transfer (HAT) mechanism as a possible explanation for the antioxidant activity of components rich in OH, NH, and SH groups, all of which contain active hydrogen atoms [33]. According to Hammad et al. (2013) and El-Kashak et al. (2017), both assays utilize a combination of HAT and electron transfer (ET) as their mechanisms [34,35]. While DPPH interacts with free OH groups in aromatic acids that possess an unsubstituted OH group, ABTS does not distinguish between phenolic OH groups. This distinction is fundamental to understanding the action mechanisms of DPPH and ABTS. Our findings indicated that the primary components in the MeOH extract of *A. judaica* were oxygenated compounds, contributing to its antioxidant properties [36]. Additionally, phytocomponents such as 2-ethylhexanoic acid, 5-hydroxy-6-(1-hydroxyethyl)-2,7-dimethoxynaphthoquinone, piperitone, and octanoic acid may play a role in the free radical scavenging activity of the *A. judaica* methanol extract. These major compounds, along with the minor components, could act individually or synergistically [37].

Terpenoid and fatty acid compounds isolated from various plants—including Coriandrum sativum [38], Reichardia tingitana [39], Salvia officinalis [40], Cleome amblyocarpa [41], and Matthiola longipetala [42]—have been identified as powerful antioxidants with significant biological activity. The primary constituent is predominantly 2-ethylhexanoic acid, which is classified as a fatty acid, along with its derivatives.

### 2.3. Allelopathic Activity of A. judaica

**Figure 2:**
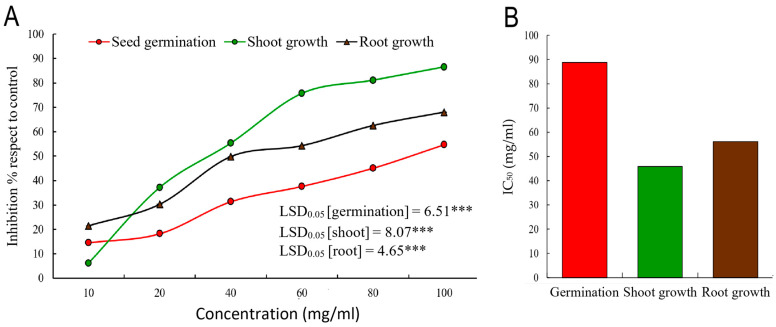
Allelopathic activity of the MeOH extract of *Artemisia judaica*. (**A**) Effect of different concentrations on seed germination, seedling shoot growth, and seedling shoot growth, and (**B**) the IC50 for seed germination, seedling shoot growth, and seedling shoot growth. *** *p*-value < 0.05.

The allelopathic activity of the MeOH extract from *A. judaica* was evaluated for its effects on seed germination and seedling development of the weed *C. murale.* At a dosage of 100 mg/mL, seed germination of *C. murale* was reduced by 54.73%. Conversely, the development of seedling roots and shoots increased by 68.04% and 86.51%, respectively (Figure 2). The shoot exhibited the lowest IC_50_ value (45.90 mg/mL) among the measured parameters, indicating a greater level of inhibition compared to the root development and seed germination of *C. murale*, which had IC_50_ values of 56.16 mg/mL and 88.80 mg/mL, respectively (Figure 2). Previous studies have indicated that the root system is more susceptible to allelochemicals than the shoot, as the roots are in direct contact with the extract and the cell membranes of root cells tend to be more permeable. This observation is supported by research from several authors [42,43]. The primary function of the root is to absorb water and nutrients from the surrounding environment and transport them to the shoot; thus, it is significantly influenced by the solutions it absorbs [44]. Additionally, allelochemicals can alter membrane permeability, inhibit cell division, and increase oxidative stress, ultimately disrupting the structure and function of the membrane [45,46].

The presence of key components in the MeOH extract contributes to significant allelopathic activity. These components include 2-ethylhexanoic acid, 5-hydroxy-6-(1-hydroxyethyl)-2,7-dimethoxynaphtho-quinone, piperitone, and octanoic acid [25]. These substances may act synergistically or individually as allelochemicals, disrupting the physiological and/or biochemical processes in the cells of *C. murale*.

According to studies [47,48], fatty acids and fatty acid derivatives, which are the main classes, exhibit a variety of biological functions. These functions include allelopathy, cytotoxicity, genotoxicity, antibacterial qualities, immune-modulatory, and antimutagenic effects. Furthermore, the chemical profiles of Pluchea dioscoridis, Rosmarinus officinalis, Anthemis melampodina, and Lavandula angustifolia have been noted to contain terpenes and oxygenated hydrocarbons, both of which display significant allelopathic activity [49].

### 2.4. Mosquito Bioassay of A. judaica

**Figure 3:**
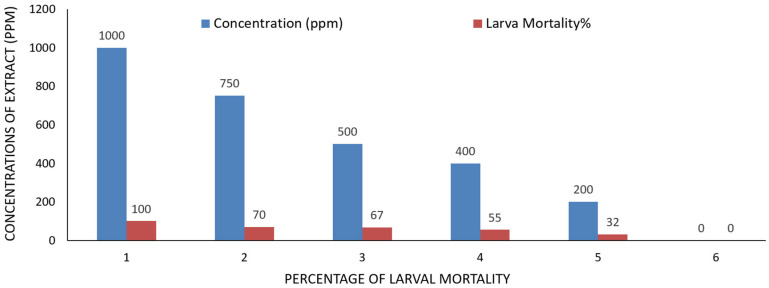
The effect of plant extract of *Artemisia judaica* on larval stages of *Aedes aegypti*.

**Figure 4:**
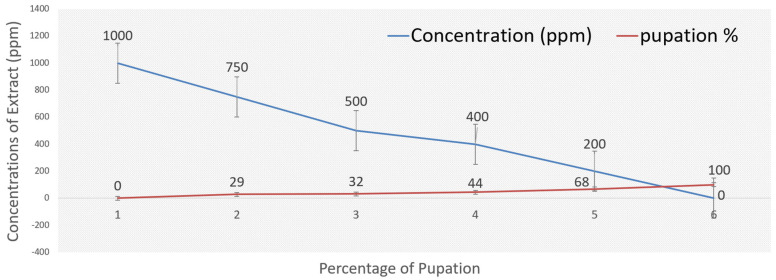
The effect of plant extract of *Artemisia judaica* on pupation percent of *Aedes aegypti*.

**Figure 5:**
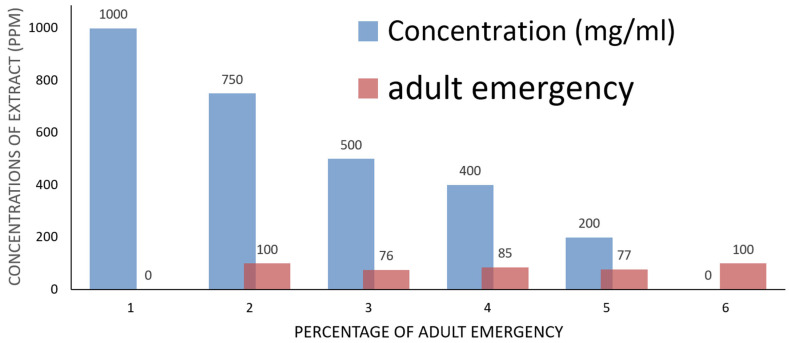
The effect of plant extract of *Artemisia judaica* on adult emergence of *Aedes aegypti* larvae.

Table 3 shows that the methanolic extract had various cytotoxic effects on *Ae. aegypti* larvae in their third instar. The highest dosage of 1000 ppm resulted in 100% larval mortality (Table 3, Figure 3). Larval death rates were observed at 70%, 67%, and 55% for doses of 750 ppm, 500 ppm, and 400 ppm, respectively. In contrast, the larval death rate at a lower concentration of 200 ppm was 32.0%, while untreated larvae (the control) showed a death rate of 0% (Table 3, Figure 3). Compared to the control, the methanol extract demonstrated non-significant toxicity at the lower concentration of 200 ppm but showed substantial toxicity at concentrations of 100 ppm and 750 ppm (Table 3). Regarding the pupation rate, the methanolic extract had similar effects; at a high concentration of 750 ppm, the pupation percentage decreased to 29.77%, whereas it increased to 68.0% at the lower concentration of 200 ppm, compared to untreated larvae (100.00 ± 0.00%) (Table 3, Figure 4). In relation to the control, the pupal death rate at 750 ppm of the methanolic extract was recorded at 00 ± 0.00%, indicating an adverse effect on pupal life (Table 3, Figure 4). Pupal mortality rates were noted at 23%, 25%, and 23% for concentrations of 500 ppm, 400 ppm, and 200 ppm, respectively. Pupal mortality did not exceed 50% at the extract doses of 750 ppm, 500 ppm, and 400 ppm, suggesting that the extract did not cause significant harm to pupal life (Table 3). Additionally, the bioactivity of the methanol extract on adult mortality and emergence was assessed. The adult emergence percentage was 77% at a concentration of 200 ppm, while it increased to 100% at 750 ppm (Table 3, Figure 5). A 30% adult death rate was recorded at 750 ppm. In comparison to untreated mosquito larvae, the adult mortality rate at 500 ppm was 20% (Table 3).

Numerous diseases transmitted by mosquitoes to both humans and animals pose significant global health challenges. *Aedes aegypti*, a small mosquito that bites during the day and is identifiable by its black and white stripes, serves as the primary vector for dengue fever. This species is also capable of transmitting various viruses to humans, including Zika, chikungunya, Mayaro, and yellow fever. Due to its prolific breeding capabilities, *Ae. aegypti* is regarded as a major global vector concern [50]. Female *Ae. aegypti* mosquitoes are responsible for spreading the virus that causes dengue, which presents clinical symptoms such as rash, bleeding, myalgia, arthralgia, headache, and retro-orbital pain. Dengue has evolved into an epidemic affecting nearly half of the world’s population, with its incidence continuing to rise annually [51].

The current study revealed that the methanol extract *of A. judaica* exhibited highly toxic effects on various life stages of the *Ae. aegypti* mosquito. At a concentration of 1000 ppm, all tested extracts resulted in a maximum larval mortality rate of 100% (refer to Table 3 and Figure 3, Figure 4 and Figure 5). At this concentration, there were no observations of pupae, adult emergence, or adult stages, as all larvae had perished. The control group showed minimal larval mortality across all periods, highlighting the strong larvicidal effects of the plant extracts. Furthermore, the standard check demonstrated significant larval mortality, confirming the reliability of our testing procedure. The observed increase in larval mortality with higher concentrations of the extracts and extended exposure times reinforces the larvicidal potential of these botanical substances. Our findings are consistent with those of Govindarajan et al. (2016), which indicated a dose-dependent response of mosquito larvae to plant extracts [52].

We support the findings of Kumar et al. (2011), which demonstrated the effectiveness of Parthenium extract in causing larval death in *A. aegypti* [53]. Our results align with Isman’s 2006 study, which suggests that the complex interactions among the bioactive components in each extract may account for differences in their efficacy [54]. This supports the notion that higher concentrations of plant extracts can disrupt *Ae. aegypti* maturation. However, not all studies have yielded the same results. Benelli et al. (2018) noted that the efficacy of plant extracts can vary by species [55]. Additionally, Ahmad et al. (2023) indicated that our findings further support the use of diverse aqueous plant extracts as insecticidal agents, offering an alternative to traditional chemical insecticides for combating *Ae. aegypti* mosquitoes [56].

## 3. Materials and Methods

### 3.1. Plant Material

The above-ground parts of *A. judaica* L. were collected from Jazan, Saudi Arabia, in May 2023, during the spring season. After collection, the specimens were placed in paper bags and transported to the laboratory. They were then crushed into a fine powder, transferred to another paper bag, and allowed to dry for one week in a covered area at a temperature of 25 °C. The identification of the plant specimens was conducted [17].

### 3.2. Preparation of Plant Extract

Thirty grams of dried plant material was ground into a fine powder and then macerated in a separate conical flask (250 mL) with 150 mL of methanol (MeOH) [6]. The mixture was then placed in a water bath shaker (Memmert WB14, Schwabach, Germany) and shaken continuously for 24 h at room temperature. German Whatman filter sheets (no. 1, 125 mm, Cat. No. 1001 125, Whatman GmbH, Dassel, Germany) were used to filter the mixture. The extraction process was conducted two more times at room temperature. The filtrates from each solvent were combined and evaporated under vacuum (HeidolphTM Instruments Hei-VAP, Heidolph Scientific Products GmbH, Schwabach, Germany) at 40 °C until all the solvent was completely removed. Once the extract was fully dried, its weight was measured, yielding 3.24 g (10.8% w/w) as a crude extract. The residue was then redissolved in methanol, and the plant extract was stored at 4 °C for future use.

### 3.3. Gas-Chromatography Mass Spectroscopy “GC-MS” Analysis

The chemical composition of *A. judaica* was determined using a well-established and validated GC-MS method [39]. The methanolic extract of the plant was analyzed using a Trace-GC-TSQ mass spectrometer (Thermo-Scientific, Austin, TX, USA), which was equipped with a direct capillary column TG-5MS (30 mm × 0.25 mm × 0.25 mm film thickness). The column’s temperature initially was set at 50 °C, then increased at a rate of 5 °C per minute until it reached 250 °C, where it remained for two minutes. Subsequently, the temperature was raised by 30 °C each minute until it reached 300 °C, at which point it held for another two minutes. Helium served as the carrier gas, flowing at a rate of 1 mL/min. After the solvent evaporated, the concentrated extract was introduced into the GC using the autosampler AS1300 (Thermo-Scientific, Austin, TX, USA).

### 3.4. Identification of Individual Components of Plant Extract

Identification of the chemical composition of each individual component extracted from the plant was obtained by comparison between their relative retention time and mass spectra data with those of the WILEY-09 and NIST.

### 3.5. Determination of Antioxidant Activity Using DPPH and ABTS Radical Scavenging Method

The antioxidant efficiency of MeOH extracts derived from the aerial parts of *A. judaica* was investigated by assessing their ability to scavenge DPPH and ABTS radicals [57].

For the DPPH assay, concentrations of 5, 10, 20, 30, 40, and 50 mg/mL of *A. judaica* extract were prepared and rapidly mixed with equal volumes of freshly prepared 0.3 mM DPPH. This mixture was incubated in the dark at 25 °C for 30 min. Absorbance was then measured at 517 nm using a spectrophotometer (Analytik Jena, Jena, Germany) [57].

In the case of the ABTS assay, the *A. judaica* MeOH extract demonstrated antioxidant activity by reducing ABTS. MeOH extract concentrations ranging from 5 to 50 mg/mL were prepared. Two mls of freshly prepared ABTS were mixed with approximately 0.2 mL of each concentration, and the resulting mixture was allowed to incubate in the dark for 6 min [57]. The absorbance at 734 nm was measured using a Spectronic 21D spectrophotometer (Milton Roy, Ivyland, CA, USA).

Ascorbic acid was used as a control, following the same protocols for the DPPH and ABTS assays at dosages of 1.0, 2.5, 5, 10, 15, and 20 mg/mL.

To determine the scavenging efficiency of both assays, the following equation was applied [12]:

(1) $$Scavenging\;efficiency\;\%=100\times (1-\frac{A\;sample}{A\;control})$$

where A sample represents the absorbance of the *A. judaica* MeOH extract, and A control represents the absorbance of the ascorbic acid.

### 3.6. Determination of Allelopathy Activity of A. judaica

#### 3.6.1. Weed Seed Source and Preparation

To evaluate the allelopathic activity of *A. judaica*, we selected the weed *Chenopodium murale* (*C. murale*) and introduced it into the cultivated area during the winter season. The seeds were collected from a farmed plot in the Kingdom of Saudi Arabia. To ensure sterility, the seeds were subjected to surface sterilization using a 0.3% sodium hypochlorite solution, immersing them for three minutes. Afterward, the seeds were rinsed three times with sterile distilled water, dried on sterilized filter paper, and stored in sterilized vials until further use [58].

#### 3.6.2. Experimental Design of Bioassay

Dimethyl sulfoxide (DMSO) was used to prepare the plant extract at various concentrations of 0.2, 0.4, 0.6, 0.8, and 1 mg/mL from the previously synthesized residues. Sterilized Whatman No. 1 filter paper was placed in 9 cm Petri dishes, each with twenty seeds. In triplicate, 5 mL of each concentration, along with DMSO as a control, was used to moisten the filter paper. To prevent contamination, the Petri dishes were placed in the growth chamber at 24 °C and covered with parafilm. The dishes were examined daily, and after ten days, the length (in mm) of the seedling roots and shoots was measured, along with the number of seeds that had germinated. The degree of inhibition in germination, root growth, and shoot growth of the seedlings was calculated as follows [59]:

(2) $$Inhibition\left(\%\right)=100\times \frac{\left(No/Length\;of\;control-No/Length\;of\;treatment\right)}{No/Length\;of\;control}$$

Using MS-EXCEL, the IC50—the concentration of the crude extract needed to inhibit seed germination or shoot growth by 50%—was determined by linearly regressing the inhibition values against different extract concentrations [19].

### 3.7. Mosquitocidal Assay

To create an emulsifier and test the toxicity of the isolated crudes, two drops of Tween 80 were applied. Following that, plant extracts were prepared in different concentrations of methanol, measured in parts per million (1000 ppm, 750 ppm, 500 ppm, 400 ppm, and 200 ppm). Thirty late third instar larvae were kept in 300 mL plastic cups containing 200 mL of dechlorinated tap water until they emerged as adults [60]. The ideal conditions were maintained at 27 ± 2 °C, 70 ± 10% relative humidity, and a 12–12 light/dark cycle. Additionally, two drops of Tween 80 were added to 100 milliliters of larval water as a control. Dead larvae and pupae were collected daily, and records of larval mortality were kept. Each experiment was conducted three times.

Following treatment, the larvae’s growth was monitored every day until pupation and adult emergence. The following parameters were then used for the estimation:

Failure to respond to mechanical stimulation was a sign of larval mortality. The larval mortality percentage was calculated as follows [60]:

(3) $$\mathrm{Larval}\;\mathrm{mortality}\;\mathrm{percentage}\;=\;\left[\frac{A-B}{A}\right]\times 100$$

where A is the number of tested larvae and B is the number of pupae that were produced.

(4) $$\mathrm{Pupation}\;\mathrm{percentage}=\left[\frac{A}{B}\right]\times 100$$

where A is the number of pupae that were produced, and B is the number of larvae that were tested.

Failure to reach the adult stage was a sign of pupal death [61]. The pupal mortality percentage was calculated as follows:

(5) $$\mathrm{Pupal}\;\mathrm{mortality}\;\mathrm{percentage}\;=\;\left[\frac{A}{B}\right]\times 100$$

where A is the number of pupae that died, and B is the number of pupae produced.

(6) $$\mathrm{The}\;\mathrm{adult}\;\mathrm{emergence}\;\%=\left[\frac{A}{B}\right]\times 100$$

where A is the number of adults who have emerged, and B is the number of pupae produced.

### 3.8. Statistical Analysis

Costat (CoHort software, version 6.4, Monterey, CA, USA) was used to assess antioxidant and allelopathic activities. The assays were conducted three times with three replications for each test. A one-way ANOVA was used to examine the statistical significance of changes in the samples.

## 4. Conclusions

The methanol extract of *A. judaica*, gathered in the Kingdom of Saudi Arabia, was obtained and analyzed using GC-MS to assess its biological activity. A total of 22 components were identified from the MeOH extract of the aerial parts of *A. judaica*. The three main constituents identified are 2-ethylhexanoic acid, 5-hydroxy-6-(1-hydroxyethyl)-2,7-dimethoxynaphtho-quinone, and piperitone, which are the most prevalent components. According to the chemical data, fatty acids and their derivatives comprise the primary category, accounting for 45.54% of the total area. The hydrocarbon class represents 30.25%, while terpenes account for 24.21%. The MeOH extract demonstrated strong antioxidant protection against free radicals, such as DPPH and ABTS, when compared to the standard antioxidant ascorbic acid. The extract also significantly inhibited the germination, shoot growth, and root growth of the weed *C. murale*. This study indicates that the methanolic extract of *A. judaica* exhibits promising larvicidal action against *Ae. aegypti* larvae, with a latent effect on their maturation into adulthood. Consequently, it may be valuable in vector management programs for mosquito control. The primary compounds responsible for these biological activities are the fatty acids and their derivatives, which may act independently or synergistically with other minor components.

## Figures and Tables

**Table 1:** The volatile components of *A. judaica* extracted with methanol.

No.	Chemical Name	Classification	Rt	Molecular Weight	Molecular Formula	RA%
	**Oxygenated hydrocarbons**					
1	Glycerol	Alcohol	10.39	92	C_3_H_8_O_3_	7.09
2	3,5-bis(1,1-dimethylethyl)-Phenol	Phenol derivatives	17.47	206	C_14_H_22_O	1.74
3	5-Hydroxy-6-(1-hydroxyethyl)-2,7-dimethoxynaphtho-quinone	Naphthoquinone	17.73	278	C_14_H_14_O_6_	17.54
4	7(E)-Tetradecenol	Fatty alcohol	25.5	212	C_14_H_28_O	0.69
5	Reynosin	Sesquiterpene lactone of the eudesmanolide group	33.7	248	C_15_H_20_O_3_	1.71
6	1,25-Dihydroxyvitamin D3	Vitamin	56.66	416	C_27_H_44_O_3_	0.84
7	Ethyl iso-allocholate	Steroids	57.3	436	C_26_H_44_O_5_	0.64
	**Fatty acids and fatty acid derivatives**					
8	Octanoic acid	Fatty acid	7.14	144	C_8_H_16_O_2_	6.30
9	2-ethylhexanoic acid	Fatty acid	8.34	144	C_8_H_16_O_2_	28.41
10	Undecane	Fatty acid derivatives	17.08	156	C_11_H_24_	0.32
11	2,5-Octadecadiynoic acid, methyl ester	Fatty acid derivatives	18.54	290	C_19_H_30_O_2_	0.52
12	Hexadecanoic acid, methyl ester	Fatty acid derivatives	27.80	270	C_17_H_34_O_2_	0.97
13	9(Z)-Octadecenoic acid, methyl ester	Fatty acid derivatives	31.77	296	C_19_H_36_O_2_	0.72
14	Methyl 4,6-tetradecadiynoate	Fatty acid derivatives	33.64	234	C_15_H_22_O_2_	4.35
15	Methyl 5,7-hexadecadiynoate	Fatty acid derivatives	33.78	262	C_17_H_26_O_2_	3.53
16	Tricosane	Fatty acid derivatives	46.96	324	C_23_H_48_	0.43
	**Terpenes**					
17	Piperitone	Monoterpenes	22.79	152	C_10_H_16_O	15.74
18	Thymol	Monoterpenes	24.00	150	C_10_H_14_O	0.65
19	Myrtenyl acetate	Monoterpenes	25.01	194	C_12_H_18_O_2_	2.23
20	Spathulenol	Sesquiterpenes	33.03	220	C_15_H_24_O	0.87
21	α-Santonin	Sesquiterpenes	46.82	246	C_15_H_18_O_3_	2.11
22	β-Santonin	Sesquiterpenes	47.02	246	C_15_H_18_O_3_	2.61
	**Total**					**100**
	Oxygenated hydrocarbon		30.25%			
	Fatty and Fatty acid derivatives		45.54%			
	Terpenes		24.21			

**Table 2:** DPPH and ABTS radicals scavenging activity percentage and IC_50_ values by the MeOH extract of *Artemisia judaica* and ascorbic acid as standard.

Treatment	Conc. (mg/mL)	Scavenging Activity (%)			
		DPPH (%)	IC_50_ (mg/mL)	ABTS	IC_50_ (mg/mL)
*Artemisia judaica*	5	9.97	31.82	7.65	39.93
	10	17.31		13.81	
	20	31.16		24.28	
	30	52.95		40.75	
	40	60.39		51.46	
	50	75.41		60.12	
	LSD_0.05_	3.96 ***			
	F-value	232.85			
Ascorbic acid	1	4.40	11.74	1.74	13.05
	2.5	13.62		9.96	
	5	40.34		36.68	
	10	52.88		45.22	
	15	59.17		55.51	
	20	72.32		68.66	
	LSD_0.05_	8.79 ***			
	F-value	49.87			

*** *p*-value < 0.05.

**Table 3:** The toxicity of plant extract of *Artemisia judaica* on different stages of *Aedes aegypti* mosquito.

Concs. ppm	Larva Mortality%	Pupation%	Pupal Mortality%	Adult Emergency%	Ault Mortality%
1000	100.00 ± 0.00	-----	----	----	----
750	70.23 ± 1.00	29.77 ± 3.00	00.00 ± 0.00	100.00 ± 2.00	30.00 ± 3.00
500	67.37 ± 2.00	32.63 ± 4.00	23.33 ± 6.00	76.67 ± 3.00	20.00 ± 2.00
400	55.23 ± 3.00	44.77 ± 2.00	25.00 ± 3.00	85.00 ± 5.00	15.00 ± 4.00
200	32.00 ± 6.00	68.00 ± 4.00	23.00 ± 5.00	77.00 ± 3.00	0
Control	0	100.00 ± 0.00	0	100.00 ± 0.00	0

## Data Availability

The data presented in this study are available upon request from the corresponding author.
